# Editorial: Current advances in seagrass research

**DOI:** 10.3389/fpls.2023.1196437

**Published:** 2023-04-17

**Authors:** Jutta Papenbrock, Mirta Teichberg

**Affiliations:** ^1^Institute of Botany, Faculty of Natural Sciences, Leibniz University Hannover, Hannover, Germany; ^2^The Ecosystems Center, Marine Biological Laboratory, Woods Hole, MA, United States

**Keywords:** seagrass, biotic and abiotic stress, genomics, seedlings, development, seagrass restoration

Seagrasses are of great ecological importance, forming large “meadows” in all continents except Antarctica and providing vital ecosystem services including primary production, carbon storage, nutrient cycling, habitat structure, and coastal protection. Seagrasses provide shelter and act as a nursery ground for commercially important small fish and invertebrates. Human activity, however, is having profound impacts on marine ecosystems, including seagrass communities. Over the last few decades anthropogenic changes, including reduced water quality, increased temperature, increased sediment loads, and higher grazing pressure, have caused global declines in seagrass populations and the area coverage of seagrass beds. Due to the valuable ecosystem services that seagrasses provide along coastlines all over the world, strategies to increase recovery of seagrass meadows are being developed; however, further research on seagrass distribution, responses to abiotic and biotic stressors and how that impacts the recovery process, acclimation or adaptation potential, and resilience to environmental change is required to support these strategies. For many regions, the exact distribution and coverage of seagrass are not well known, and simple methods to reliably detect changes in seagrass coverage need to be established for the development of locally successful conservation methods. This Research topic, therefore, aimed to advance seagrass research by bringing together different perspectives on seagrasses that highlight their ecological importance and the effects of anthropogenic pressures, as well as the potential for its recovery and restoration.

Some very basic seagrass physiological research questions have been addressed, partially with the help of genomics. It was found that the increasing degradation rate of the foundation species *Phyllospadix iwatensis* may be related to the intrinsic photosensitivity of the photosystem II oxygen-evolving complex. Ambient harsh light conditions inactivate this complex irreversibly contributing to the low resilience of this species to light stress (Wang et al.).

Knowledge of seed development in seagrasses, the only group of angiosperms to complete their life cycle submerged in marine environments, is still very limited. The regulation of the energy metabolism during the seed-to-seedling transition in *Zostera marina* was investigated by integrated metabolomic, transcriptomic, and physiological analysis (Zhu et al.). One of the main findings showed that the TCA cycle is the most effective pathway to supply energy for germination and seedling growth through the upregulation of a large number of genes and metabolites, whereas the pentose phosphate pathway plays a supplementary role during seedling establishment.

Among the most basic seagrass traits are those associated with the salt tolerance mechanism of seagrasses, essential for surviving in the marine environment. The physiological basis of salt tolerance was investigated in *Thalassia hemprichii* and substantiated with gene expression analysis (Shen et al.). The optimal salinity for *T. hemprichii* is 25 to 35 PSU. Shen et al. found that gene expression changed rapidly upon exposure to salinity stress. The differentially expressed genes regulate transport and metabolism by promoting environmental adaptation.

Another relevant adaptation factor to the saline environment is the unique composition of the cell wall of seagrasses. A study of the composition of the cell wall revealed a new combination of structural polysaccharides, such as polyanionic, low-methylated pectins, and glycoprotein elements, such as arabinogalactan proteins (AGPs) originating from both macroalgae and angiosperm land plants (Pfeifer and Classen).

As a biotic stressor, increased grazing intensity due to climate change may inhibit or prolong the recovery time of a colonizing seagrass (*Halophila ovalis*) with implications for seagrass resilience (O’Dea et al.). O’Dea et al. found that a higher grazing intensity by waterfowl on seagrasses took much longer to recover than minimal grazing intensity. Recovery times were also more variable with increased grazing intensity. Most of the recovery was due to vegetative rather than reproductive growth.

There may also be significant indirect effects of grazing within seagrass meadows. Mesograzers which often graze on epiphytic communities of seagrasses help to minimize the effects of the overgrowth of algae. However, when seagrass ecosystems are spatially connected to other macrophyte communities, such as large kelp forests, grazers preferentially choose kelp over epiphytes of seagrass (Olson et al.), indicating that allochthonous sources may be more important than previously assumed. This has implications for trophic structure and possibly could diminish the role of mesograzers in the alleviation of competition between seagrasses and their epiphytes for light.

Another example of biotic interaction that challenges seagrass ecosystems is the introduction of invasive species. The invasive salt marsh halophyte *Spartina alterniflora* was found growing further into the intertidal zone in the Yellow River Delta, China, where it threatened the habitat of the native seagrass *Zostera japonica* (Yue et al.).

However, there are also examples of the positive effects of interactions. Nested interactions between chemosynthetic lucinid bivalves and seagrass promote ecosystem functioning in contaminated sediments (Cardini et al.).

Successful approaches to restoring seagrass meadows are needed to promote recovery. Evidence was provided that the addition of nutrients to seagrass seed planting improved seedling emergence and doubled maximum shoot length, even in nutrient-rich environments (Unsworth et al.). These sources of nutrients allow for the establishment of new seedlings in their environment.

Light is one of the most important abiotic factors limiting seagrass distribution and growth. Reduction of light caused by water quality and increased sediment loads leads to seagrass decline. Xu et al. tested a novel method of suspending seagrasses in floating rafts at varying depths of the water column and measuring seagrass responses over time in Ailian Bay, Northern China, and found that over longer periods of time, seagrass highest shoot densities and reproducing shoots were found only in the shallowest water depths. This is important to determine the maximum depth at which transplants will survive, grow, and reproduce at the specific locality prior to transplantation attempts.

Reciprocal field transplantation experiments with *Zostera marina* L. revealed differences in seed germination time of two populations from different geographic regions. The analyses were supported by comparative transcriptome analysis indicating the power of combining field experiments with molecular methods (Zhang et al.).

A review paper by Nguyen et al. highlighted the current advances in seagrass research from one country, Vietnam. One goal of the review is to support decision-makers in developing science-based conservation strategies. An alarming decline in the coverage of seagrass meadows in almost all parts of Vietnam is observed. Since 1990 a decline of 46.5% or 13,549 hectares was found. Only in a few protected and/or difficult-to-reach areas was an increase observed. Conditions at those sites should be investigated in more detail to make suggestions for the conservation and recovery of seagrass meadows. Only decisions based on the interdisciplinary cooperation of scientists from all disciplines mentioned will finally lead to conserving this valuable ecosystem for humanity and biodiversity.

Another review more broadly explored the use of a trait-based approach to seagrass ecology, an approach that has been extensively applied in terrestrial plant research. Although morphological and physiological trait data have been collected for other purposes, until more recently, seagrass trait-based approaches have been minimally used. This provides an opportunity to re-explore these data with a trait-based approach. Moreira-Saporiti et al. provide a framework for exploring hypotheses linking seagrass trait responses to the environment and to ecosystem functions and services using a trait-based approach.

Many new research questions were explored by this Research Topic, yet even more questions remain open. We still need to develop non-invasive methods to better understand the spatial and temporal decline and recovery of seagrass meadows all over the world. There are still knowledge gaps in understanding the functioning and resilience of seagrass ecosystems and trophic interactions under global change. Filling these gaps would help pave the way for developing methods of seagrass restoration and have implications for their use as nature-based solutions.

## Author contributions

All authors listed have made a substantial, direct, and intellectual contribution to the work and approved it for publication.

